# Hypoxia-induced 26S proteasome dysfunction increases immunogenicity of mesenchymal stem cells

**DOI:** 10.1038/s41419-019-1359-x

**Published:** 2019-01-28

**Authors:** Ejlal Abu-El-Rub, Glen Lester Sequiera, Niketa Sareen, Weiang Yan, Meenal Moudgil, Mohammad Golam Sabbir, Sanjiv Dhingra

**Affiliations:** 1 Regenerative Medicine Program, Institute of Cardiovascular Sciences, Department of Physiology and Pathophysiology, University of Manitoba, Winnipeg, MB Canada; 2 St. Boniface Hospital Albrechtsen Research Centre, Winnipeg, MB Canada; 3 Division of Neurodegenerative Disorders, University of Manitoba, Winnipeg, MB Canada

## Abstract

Bone marrow-derived allogeneic (donor derived) mesenchymal stem cells (MSCs) are immunoprivileged and are considered to be prominent candidates for regenerative therapy for numerous degenerative diseases. Even though the outcome of initial allogeneic MSCs based clinical trials was encouraging, the overall enthusiasm, of late, has dimmed down. This is due to failure of long-term survival of transplanted cells in the recipient. In fact, recent analyses of allogeneic MSC-based studies demonstrated that cells after transplantation turned immunogenic and were subsequently rejected by host immune system. The current study reveals a novel mechanism of immune switch in MSCs. We demonstrate that hypoxia, a common denominator of ischemic tissues, induces an immune shift in MSCs from immunoprivileged to immunogenic state. The immunoprivilege of MSCs is preserved by downregulation or the absence of major histocompatibility complex class II (MHC-II) molecules. We found that 26S proteasome-mediated intracellular degradation of MHC-II helps maintain the absence of MHC-II expression on cell surface in normoxic MSCs and preserves their immunoprivilege. The exposure to hypoxia leads to dissociation of 19S and 20S subunits, and inactivation of 26S proteasome. This prevented the degradation of MHC-II and, as a result, the MSCs became immunogenic. Furthermore, we found that hypoxia-induced decrease in the levels of a chaperon protein HSP90α is responsible for inactivation of 26S proteasome. Maintaining HSP90α levels in hypoxic MSCs preserved the immunoprivilege of MSCs. Therefore, hypoxia-induced inactivation of 26S proteasome assembly instigates loss of immunoprivilege of allogeneic mesenchymal stem cells. Maintaining 26S proteasome activity in mesenchymal stem cells preserves their immunoprivilege.

## Introduction

Bone marrow-derived mesenchymal stem cells (MSCs) are considered to be immunoprivileged, because these cells do not express or have negligible expression of cell surface immune antigen—major histocompatibility complex class II (MHC-II) molecules^1,2^. The MHC-II molecules are cell surface immune antigens that provide signals to alert the host immune system to initiate immune response against transplanted cells^3^. Owing to negligible expression or the absence of MHC-II on the surface of MSCs, transplanted allogeneic MSCs (donor derived) are able to escape the recipient’s immune system and survive in the host. These unique properties have made allogeneic MSCs the flagbearer of regenerative medicine. In several animal models of degenerative diseases including neurodegenerative, cardiovascular, and autoimmune disorders, the transplanted allogeneic MSCs were able to initiate repair processes and improve function^4–7^. Based on the encouraging outcome of preclinical studies, several clinical trials have been conducted to assess the safety and efficacy of allogeneic MSCs^8^. Even though the outcome of initial animal studies and clinical trials was positive, but the overall enthusiasm, of late, has dimmed down. This is due to failure of long-term survival of transplanted cells and diminishing benefits over a period of time after transplantation. In fact, the recent data from preclinical studies and clinical trials indicate that allogeneic MSCs after transplantation provoke an immune response in the recipient^9–12^. In a pig model, allogeneic MSCs elicited immune responses after transplantation in the ischemic heart^10^. We recently reported in a rat model of myocardial infarction that allogeneic MSCs after 5 weeks of transplantation became immunogenic and were rejected in the infarcted/ischemic heart^12^. These findings strongly suggest that allogeneic MSCs become immunogenic after implantation in the ischemic tissues in recipient and are rejected by host immune system. Therefore, understanding the mechanisms of immune switch in MSCs from immunoprivileged to immunogenic state would help in planning strategies to prevent rejection and enhance benefits of allogeneic MSC-based therapy. Hypoxia (part of ischemic environment) is a harsh hallmark of many pathological diseases including cardiovascular diseases^13–16^. In this study, we examined the effect of hypoxic environment on the immunoprivilege of MSCs. Our studies reveal that exposure to hypoxic conditions instigates an immune switch in MSCs from immunoprivileged to immunogenic state. The current study also provides a novel mechanism of hypoxia-induced immune switch in MSCs.

## Results

### Exposure to hypoxic environment triggers loss of immunoprivilige in MSCs

**Fig. 1:**
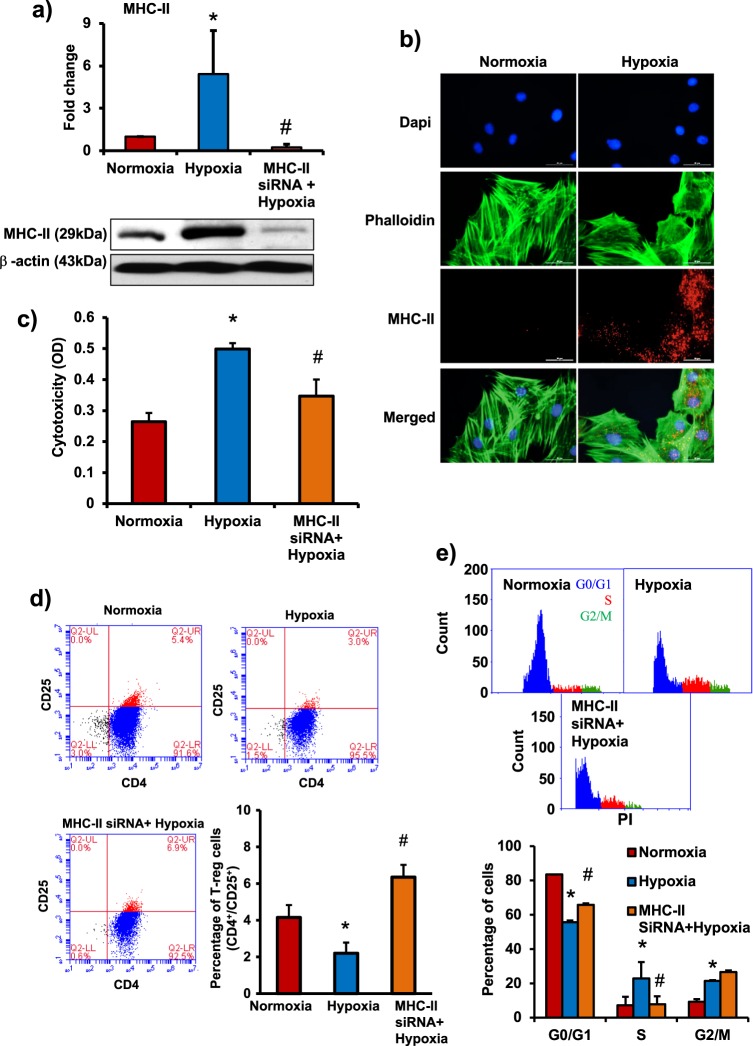
Exposure to hypoxia induces loss of immunoprivilege in MSCs. **a** Rat bone marrow-derived MSCs were exposed to hypoxia for 24 h. MHC-II levels as measured by western blotting increased in hypoxic MSCs, which showed regression when inhibited by siRNA. *n* = 3. **b** Immunofluorescence images showed a significant increase in the expression of MHC-II under hypoxia compared with normoxia. *n* = 6. **c**–**e** To determine the immunogenicity of MSCs, normoxic and hypoxic rat MSCs (with or without siRNA) were co-cultured with allogeneic leukocytes at a ratio 1:10 for 72 h. **c** Leukocyte-mediated cytotoxicity in MSCs (LDH release) increased significantly in hypoxic MSCs vs. normoxic cells, which was rescued by siRNA-mediated inhibition of MHC-II. *n* = 10. **d** The effect of MSCs on Treg cell (CD4^+^CD25^+^) induction in a mixed leukocyte population was assessed by flow cytometry. The number of Treg cells decreased after co-culture with hypoxic MSCs, siRNA-mediated inhibition of MHC-II increased Treg cell number. *n* = 3. **e** The effect of MSCs on leukocyte activation and proliferation was determined using PI staining, by assessing the number of cells present in different phases of cell cycle. The % of activated and proliferating leukocytes showed a significant increase under hypoxia. The number of activated and proliferating leukocytes decreased after siRNA-mediated MHC-II inhibition in MSCs. *n* = 3. *p < 0.05 compared with normoxia group; #*p* < 0.05 compared with hypoxia group. Each experiment was repeated four to six times

Immunoprivilege of MSCs is preserved by the absence of MHC-II molecules^1,2^. We wanted to determine whether there was any change in the expression of MHC-II in MSCs under hypoxic conditions. BM-MSCs were incubated in the hypoxia chamber for 24 h, MHC-II levels were assessed by western blotting and immunostaining. There was a significant increase in MHC-II levels in hypoxia-exposed MSCs compared with normoxic cells (Fig. 1a, b).

To investigate association between hypoxia-induced MHC-II upregulation and immunogenicity of MSCs, the stem cells were co-cultured with allogeneic leukocytes for 72 h and the extent of leukocyte-mediated cytotoxicity in MSCs was measured. The cytotoxicity was measured by determining the amount of lactate dehydrogenase (LDH) released and was found to be significantly greater in hypoxic MSCs compared with normoxic cells (Fig. 1c). Interestingly, small interfering RNA (siRNA)-mediated inhibition of MHC-II prevented leukocyte-mediated cytotoxicity in hypoxic MSCs (Fig. 1a, c). Therefore, we infer that hypoxia-induced increase in MHC-II levels is associated with loss of immunoprivilege of MSCs. However, the presence of siRNA against MHC-II did not change the level of cytotoxicity in normoxic MSCs after co-culture with allogeneic leukocytes (Supplementary Figure 1). MSCs are immunoprivileged and promote immune tolerance by enabling the phenotype change from cytotoxic T cells toward regulatory T (Treg) cell population^17,18^. Treg cells can suppress the proliferation of cytotoxic T cells and promote immune tolerance. In the current study, we counted the number of CD4^+^CD25^+^ Treg cells in a mixed leukocyte population after 72 h of co-culture with allogeneic MSCs by flow cytometry. The Treg cell number decreased after co-culture with hypoxia-exposed MSCs compared with normoxic cells (Fig. 1d). MHC-II-inhibited MSCs were able to promote Treg cell induction (Fig. 1d).

MSCs also have the ability to suppress leukocyte proliferation and promote immune tolerance^19^. The leukocyte activation and proliferation was measured by counting the number of cells entering S-phase and G2/M phase from G0/G1 phase of the cell cycle and by cell proliferation assay kit. There was a significant increase in leukocyte proliferation after co-culture with hypoxic MSCs compared with normoxic cells (Fig. 1e, Supplementary Figure 2). The number of resting leukocytes in G0/G1 phase were greater after co-culture with normoxic MSCs compared with hypoxic MSCs (Fig. 1e). At the same time the number of leukocytes entering S-phase (proliferating phase) and G2M phase increased after co-culture with hypoxic MSCs compared with normoxic stem cells (Fig. 1e). These results demonstrate that MSCs under normoxic conditions were able to suppress leukocyte proliferation, after exposure to hypoxia, MSCs lost this ability. The co-culture with MHC-II-inhibited hypoxic MSCs decreased leukocyte proliferation, as there was an increase in the leukocyte number in G0/G1 phase and decrease in the leukocyte number in S-phase (Fig. 1e, Supplementary Figure 2). Therefore, hypoxia-induced upregulation of MHC-II was associated with increase in immunogenicity and a decrease in immune tolerance of allogeneic MSCs.

### 26S proteasome degrades MHC-II in normoxic MSCs and preserves immunoprivilege

**Fig. 2:**
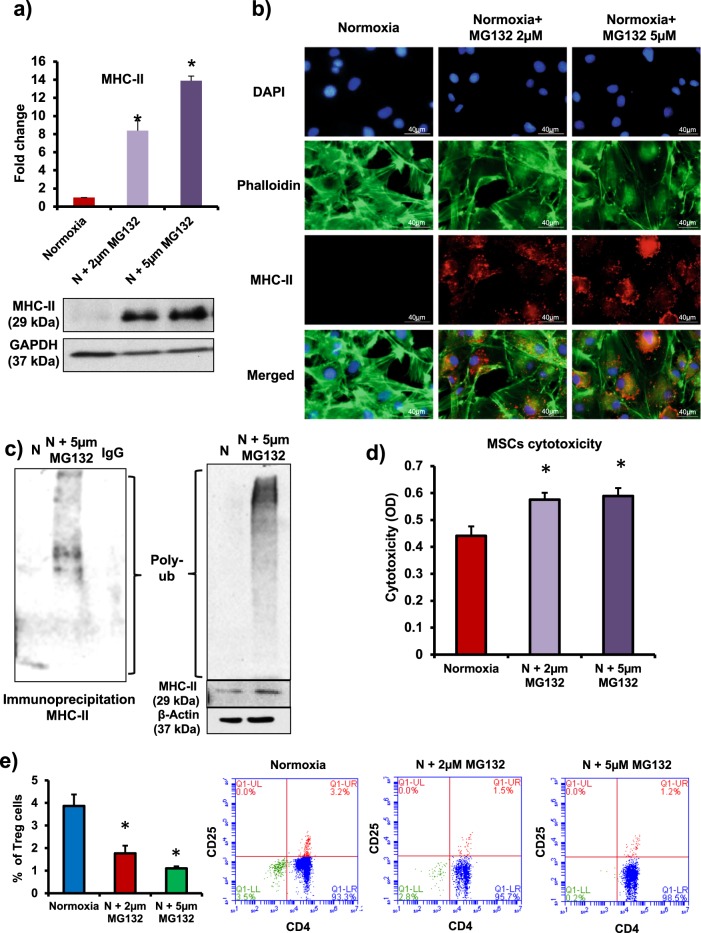
26S proteasome regulates MHC-II levels and preserves immunoprivilege of MSCs. **a**, **b** Rat MSCs were treated with 26S proteasome inhibitor (MG132, 2 µM and 5 µM for 24 h). MHC-II levels determined by western blotting (**a**) and immunostaining (**b**) showed a dose-dependent increase (*n* = 3). **c** Immunoprecipitation (IP) analysis was performed in rat MSCs with or without 26S inhibitor to determine the involvement of 26S proteasome in the degradation of MHC-II. IP data revealed a significant accumulation of ubiquitinated MHC-II protein in 26S-inhibited group. IP was performed with MHC-II antibody and blotting was performed with polyubiquitin antibody. Left panel: IP; right panel: lysate (*n* = 4). **d**, **e** To determine the immunogenicity of MSCs, normoxic MSCs (with or without 26S inhibitor) were co-cultured with allogeneic leukocytes at a ratio 1:10 for 72 h. **d** LDH levels increased significantly in 26S inhibitor-treated MSCs (*n* = 10). **e** Treg (CD4^+^CD25^+^) cell number in the mixed leukocyte population decreased significantly after co-culture with 26S inhibited MSCs *n* = 3. *p < 0.05 compared with normoxia group. Each experiment was repeated four to six times

Next, we wanted to determine the mechanisms that lead to the absence of MHC-II in normoxic MSCs. Intracellular synthesis, activation, transport, and storage of MHC-II have been studied extensively, but the turnover of MHC-II protein itself remains largely unexplored. In this regard, 26S proteasome system is reported to mediate degradation of unwanted or damaged proteins by proteolysis^20^. Therefore, to explore the possibility of MHC-II degradation by 26S proteasome system in normoxic MSCs, we incubated the cells with 26S proteasome inhibitor, MG132 (2 µM and 5 µM) for 24 h followed by determination of MHC-II expression. There was a dose-dependent increase in MHC-II protein levels in normoxic MSCs in the presence of 26S inhibitor (Fig. 2a, b). This dose and treatment protocol (for MG132 treatment) was optimal based on our pilot studies (Supplementary Figure 3).

To mediate degradation of unwanted proteins by 26S proteasome, the lysine residue of target protein (protein to be degraded) binds to ubiquitin (a small protein, 8.5 kDa) and this complex (ubiquitinated protein) is recognized by 26S proteasome that catalyzes its degradation and clearance. We performed immunoprecipitation (IP) assay to monitor the levels of ubiquitinated MHC-II in normoxic MSCs before and after treating the cells with 26S inhibitor. There was a significant increase in the accumulation of ubiquitinated MHC-II in MG132-treated MSCs (Fig. 2c). Interestingly, we also found a significant increase in the accumulation of ubiquitinated MHC-II in hypoxia-treated MSCs compared with normal stem cells (Supplementary Figure 4). These data suggest that in normoxic MSCs, 26S proteasome facilitates degradation of MHC-II and pharmacological inhibition of 26S proteasome or exposure to hypoxia lead to increase in MHC-II levels in MSCs. Furthermore, in the MSCs and allogeneic leukocytes co-culture experiment, the presence of 26S inhibitor increased leukocyte-mediated cytotoxicity in normoxic MSCs (Fig. 2d). However, siRNA-mediated inhibition of MHC-II in MG132-treated normoxic MSCs prevented the leukocyte-mediated cytotoxicity (Supplementary Figure 1). Therefore, upregulation of MHC-II in 26S inhibitor-treated MSCs is responsible for leukocyte-mediated cytotoxicity. In our co-culture experiments we also found that the number of CD4^+^CD25^+^ Treg cells decreased and leukocyte proliferation increased after co-culture with 26S inhibited MSCs compared with normoxic cells (Fig. 2e, Supplementary Figure 5). Therefore, 26S proteasome-mediated degradation of MHC-II preserves immunoprivilege of normoxic MSCs.

### Exposure to hypoxia leads to inactivation of 26S proteasome assembly in MSCs

**Fig. 3:**
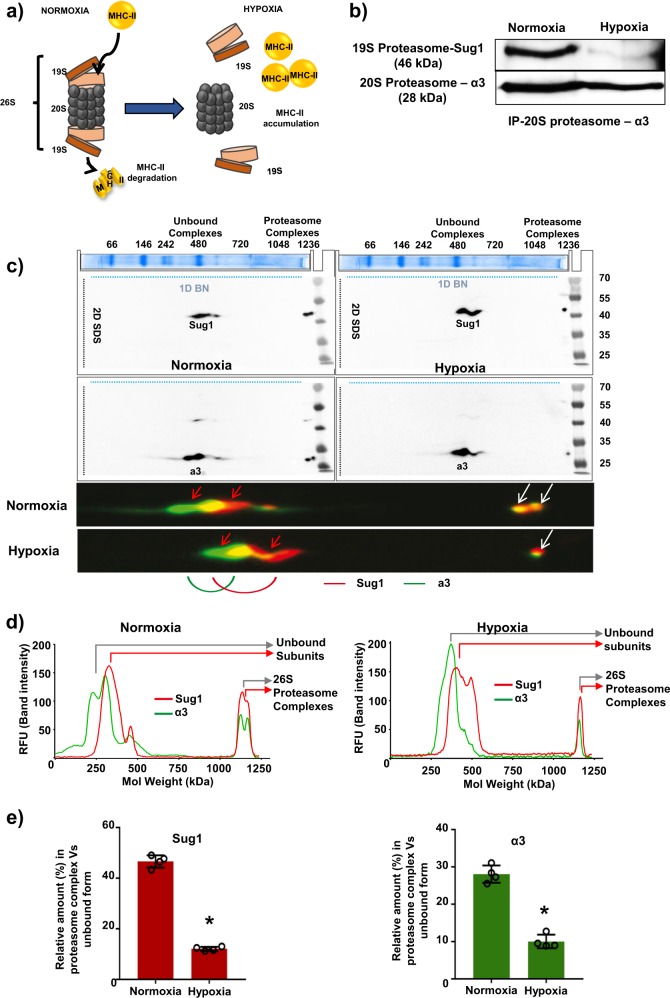
Exposure to hypoxia led to dissociation of 26S proteasome complex in rat MSCs. **a** Model depicts 26S proteasome structure; MHC-II degradation by 26S maintains absence of MHC-II in normoxic MSCs. Hypoxia-induced dissociation of 26S proteasome (19S and 20S subunits) results in accumulation of MHC-II. **b** Immunoprecipitation (IP) assay was performed to monitor interaction between 19S and 20S subunits in normoxic and hypoxic MSCs. IP was performed with 20S antibody and blotted with antibodies for 19S and 20S. In normoxic MSCs, 19S and 20S subunits bind to form functional 26S proteasome. The binding of two subunits decreased in hypoxic MSCs (*n* = 4). **c** The two-dimensional (2D) blue-native polyacrylamide gel electrophoresis (BN-PAGE)/SDS-PAGE assay was performed to study protein–protein interaction between subunits of 26S proteasome. The cell lysates from normoxia- and hypoxia-exposed MSCs were subjected to 2D SDS-PAGE and immunoblotted using specific antibodies for Sug1 (one of the constituents of 19S subunit) and α3 (one of the constituents of 20S subunit). The multiprotein complex appearing in the high molecular weight range ~1200–2000 kDa (white arrows) represent 26S proteasome. The amount of 26S complex was lesser in hypoxia-exposed cells compared with normoxic group (*n* = 3). **d** Quantitative densitometric analysis of 2D immunoblots reveal that the fluorescence intensity (RFU) of 26S complex is stronger in normoxic MSCs compared with hypoxia-exposed cells (*n* = 3). **e**The ratio of bound vs. unbound fractions of Sug1 (19S subunit) and α3 (20S subunit) involved in the formation of 26S proteasome complex were significantly higher in normoxic cells compared with hypoxia-exposed MSCs (*n* = 3). *p < 0.05 compared with normoxic MSC. Each experiment was repeated three to four times

The inhibition of 26S proteasome activity in normoxic MSCs was associated with loss of immunoprivilege. Also, there was a significant increase in the accumulation of ubiquitinated MHC-II in MSCs under hypoxia (Supplementary Figure 4). These findings prompted us to test that the observed MHC-II upregulation and loss of immunoprivilege during hypoxia might be related to decreased 26S function and activity. The 26S proteasome activity requires binding as well as coordinated action of 19S and 20S subunits for carrying out degradation and proteolysis of ubiquitinated proteins (Fig. 3a). We performed IP assay to assess the binding of 19S proteasome subunit and 20S proteasome subunit in normoxic and hypoxic MSCs. Our data demonstrate a dramatic decrease in the binding between 19S and 20S subunits in hypoxic MSCs (Fig. 3b). In order to further verify that exposure to hypoxia is associated with disassociation of 26S proteasome assembly, we performed two-dimensional (2D) blue-native polyacrylamide gel electrophoresis (BN-PAGE/SDS-PAGE) assay to study protein–protein interaction between subunits of 26S proteasome. The cell lysates from normoxia- and hypoxia-exposed MSCs were subjected to 2D SDS-PAGE and immunoblotted using specific antibodies for Sug1 (one of the constituents of 19S subunit) and α3 (one of the constituents of 20S subunit). In 2D SDS-PAGE, the first dimension “native PAGE” separates whole multiprotein complexes (MPCs) and the second dimension “denatured SDS-PAGE” separates interacting protein components within one MPC, which appears on a vertical line^21^. The Sug1 and α3 bind together only when these two proteins are part of respective 19S and 20S subunits of the 26S proteasome complex. Further, the molecular weight of functional 26S proteasome complexes has been reported to be in the range of 1200–2000 kDa^22–24^. As we used specific antibodies for Sug1 and α3 proteins for immunoblotting, therefore the MPC appearing in the high-molecular-weight range ∼1200 kDa (Fig. 3c, white arrows in the lower panel) represent 26S proteasome (molecular weight ∼1200 kDa). The remaining low-molecular-weight complexes where Sug1 and α3 appeared partially overlapped may refer to other protein complexes involving Sug1 and α3 (Fig. 3c, red arrows). Interestingly, the dynamics of 26S proteasome complex were significantly different in hypoxic MSCs vs. normoxic cells; the amount of 26S complex was lesser in hypoxia-exposed cells compared with normoxic group (Fig. 3c, white arrows). Furthermore, quantitative densitometric analysis of 2D immunoblots reveal that the fluorescence intensity Relative Fluorescence Unit (RFU) of 26S complex is stronger in normoxic MSCs compared with hypoxia-exposed cells (Fig. 3d). In addition, the ratio of bound vs. unbound fractions of Sug1 (19S subunit) and α3 (20S subunit) involved in the formation of 26S proteasome complex were significantly higher in normoxic cells compared with hypoxia-exposed MSCs (Fig. 3e). These data confirm that binding between Sug1 and α3 subunits decreased in MSCs under hypoxia.

**Fig. 4:**
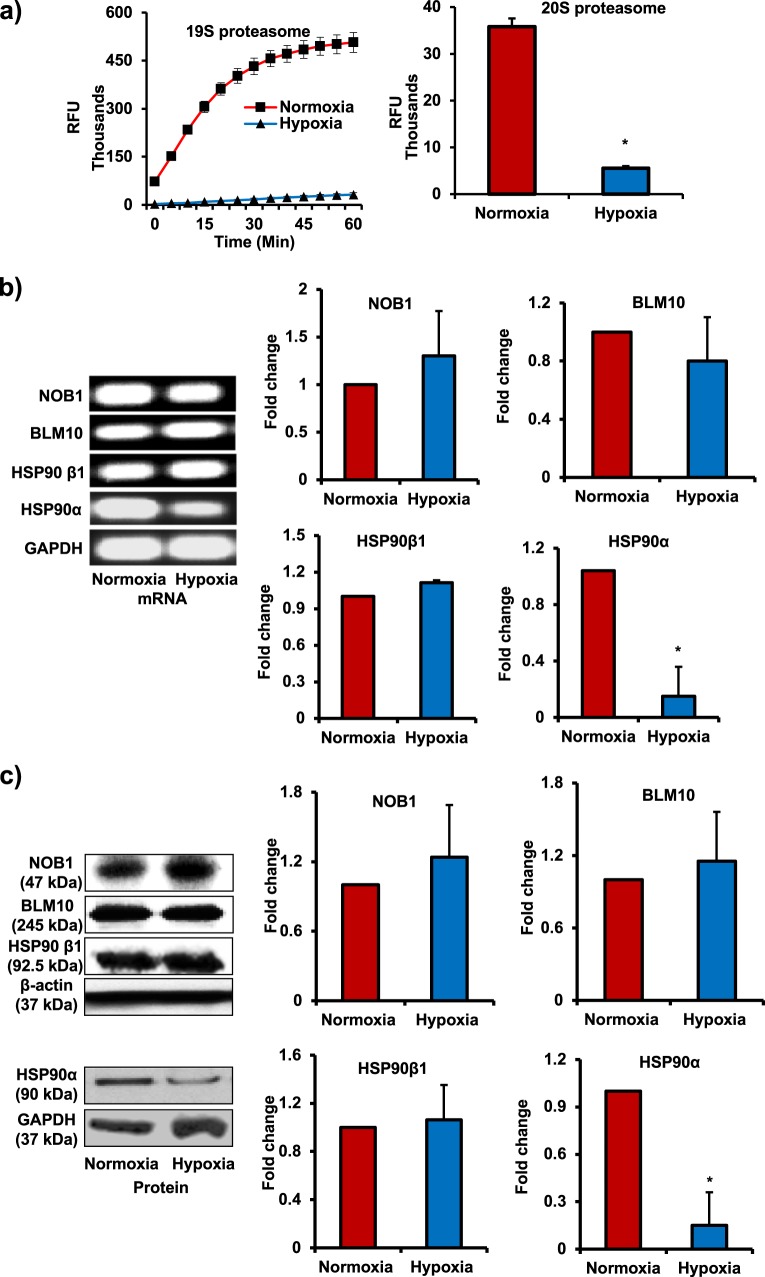
26S proteasome activity and HSP90α levels were downregulated in hypoxic rat MSCs. **a** To measure 26S activity, the levels of both 19S (deubiquitinating activity) and 20S (proteolysing activity) were determined. The activities were measured by using flurogenic substrates:- U-555 for 19S and SUC-LLVY-AMC for 20S. Hypoxic MSCs were found to have a marked reduction in 26S activity (*n* = 3). **b**, **c** NOB1, BLM10, HSP90α, and HSP90β mRNA and protein levels were determined by RT-PCR and western blotting. NOB1, BLM10, and HSP90β levels did not change in MSCs after exposure to hypoxia for 24 h. However, HSP90α mRNA and protein levels decreased in hypoxia-exposed MSCs (*n* = 4); *p < 0.05 compared with normoxic MSC. Each experiment was repeated four to six times

To mediate proteolytic actions of 26S proteasome, the 19S subunit recognizes ubiquitinated target proteins, unfolds, and translocates them to the interior of 20S subunit, where proteins finally get proteolysed^25^. Both 19S and 20S subunits are able to perform deubiquitination and proteolysis of target proteins only when these two subunits are assembled as 26S proteasome. Therefore, to precisely determine 26S activity, we measured deubiquitinating activity of 19S and proteolysing activity of 20S by fluorescence assays. There was a significant decrease in the activities of 19S and 20S proteasomes in hypoxic MSCs compared with normoxic cells (Fig. 4a). These studies demonstrate that exposure to hypoxia led to dissociation and inactivation of 26S proteasome assembly in MSCs compared with normoxic cells.

### Hypoxia-induced downregulation of a chaperon protein heat shock protein 90α leads to dissociation of 26S proteasome and loss of immunoprivilege of MSCs

Molecular chaperones including bleomycin resistance protein 10 (BLM10), heat shock protein 90α (HSP90α), HSP90β, and NIN1-binding protein 1 (NOB1) are reported to have a role in assembling and maintenance of 26S proteasomal machinery^25,26^. Alterations in chaperone protein levels result in defective assembling or dissociation of 19S and 20S complex that affects the proteolytic function of 26S. To understand the mechanisms of hypoxia-induced dissociation of 26S proteasome, we measured the levels of these chaperons in MSCs before and after hypoxia treatment. Interestingly, the messenger RNA and protein levels of BLM10, HSP90β, and NOB1 did not change significantly in MSCs under hypoxia (Fig. 4b, c). However, we found a significant decrease in both mRNA and protein levels of HSP90α in hypoxic MSC (Fig. 4b, c).

**Fig. 5:**
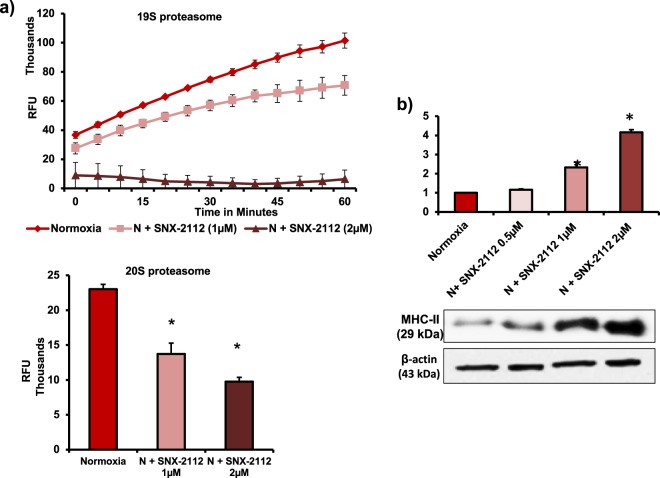
HSP90α regulates 26S activity and MHC-II levels in normoxic MSCs. **a**, **b** Rat MSCs were treated with HSP90α inhibitor (SNX-2112, 0.5 µM, 1 µM, and 2 µM for 24 h), 26S activity (19S and 20S activities) by fluorescence assay and MHC-II levels by western blotting were measured. **a** 26S activity decreased in HSP90α-inhibited MSCs (*n* = 3). **b** MHC-II expression increased in HSP90α-inhibited MSCs in a dose-dependent manner (*n* = 4). *p < 0.05 compared with normoxic MSC. Each experiment was repeated four to six times

In the next set of experiments, we wanted to investigate whether HSP90α regulates 26S proteasome activity in normoxic MSCs and hypoxia-induced decrease in HSP90α was associated with the inactivation of 26S proteasome system and increase in immunogenicity of MSCs. We blocked HSP90α in normoxic MSCs using pharmacological inhibitor (SNX-2112) and measured deubiquitinating activity of 19S and proteolysing activity of 20S by fluorescence assays. There was a significant decrease in the activities of 19S and 20S subunits in HSP90α-inhibited normoxic MSCs (Fig. 5a). We also found a dose-dependent increase in MHC-II levels in HSP90α-inhibited normoxic MSCs (Fig. 5b).

**Fig. 6:**
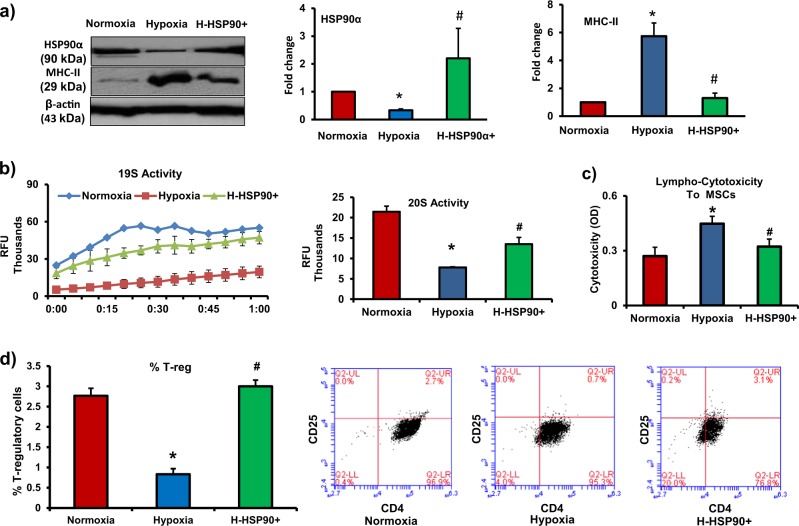
Maintaining HSP90α levels preserves immunoprivilege of MSCs under hypoxia. **a** Rat MSCs were transduced with lentiviral construct to overexpress HSP90α. HSP90α and MHC-II levels were measured by western blotting in normoxic, hypoxic, and HSP90α-overexpressing hypoxic MSCs (*n* = 4). **b** 26S proteasome activity by fluorescence assay in normoxic, hypoxic, and HSP90α-overexpressing hypoxic MSCs. HSP90α overexpression rescued 26S activity in hypoxic MSCs (*n* = 4). **c**, **d** To determine the immunogenicity of MSCs, normoxic MSCs, hypoxic MSCs, and HSP90α-overexpressing hypoxic MSCs were co-cultured with allogeneic leukocytes at a ratio 1:10 for 72 h. **c** LDH levels increased significantly in hypoxic MSCs, HSP90α overexpression prevented hypoxia-induced increase in LDH levels (*n* = 10). **d** Treg (CD4^+^CD25^+^) cell number in the mixed leukocyte population decreased significantly after co-culture with hypoxic MSCs; HSP90α-overexpressing hypoxic MSCs were able to induce Treg cell number. *n* = 3. Each experiment was repeated four to six times. *p < 0.05 compared with normoxia group; #*p* < 0.05 compared with hypoxia group

To assess whether maintaining HSP90α in hypoxic MSCs would preserve immunoprivilege of stem cells, we used lentiviral particles to over express HSP90α in MSCs. The lentivirus-mediated overexpression of HSP90α maintained the levels of HSP90α in hypoxic MSCs (Fig. 6a). We employed same polyvinylidene difluoride (PVDF) membrane to probe it with MHC-II antibody; our data demonstrate that maintaining HSP90α levels prevented hypoxia-induced increase in MHC-II levels (Fig. 6a). We also found that maintaining HSP90α prevented hypoxia-induced downregulation of 26S activity (Fig. 6b). Furthermore, overexpression of HSP90α decreased -mediated cytotoxicity in hypoxic MSCs (Fig. 6c). In allogeneic MSCs and leukocyte co-culture experiments, HSP90α-overexpressing hypoxic MSCs were able to increase Treg cell numbers in mixed leukocyte population (Fig. 6d). Therefore, maintaining HSP90α levels in MSCs prevented hypoxia-induced decrease in 26S activity and preserved their immunoprivilege.

### Exposure to hypoxia leads to loss of immunoprivilege in human BM-MSCs

**Fig. 7:**
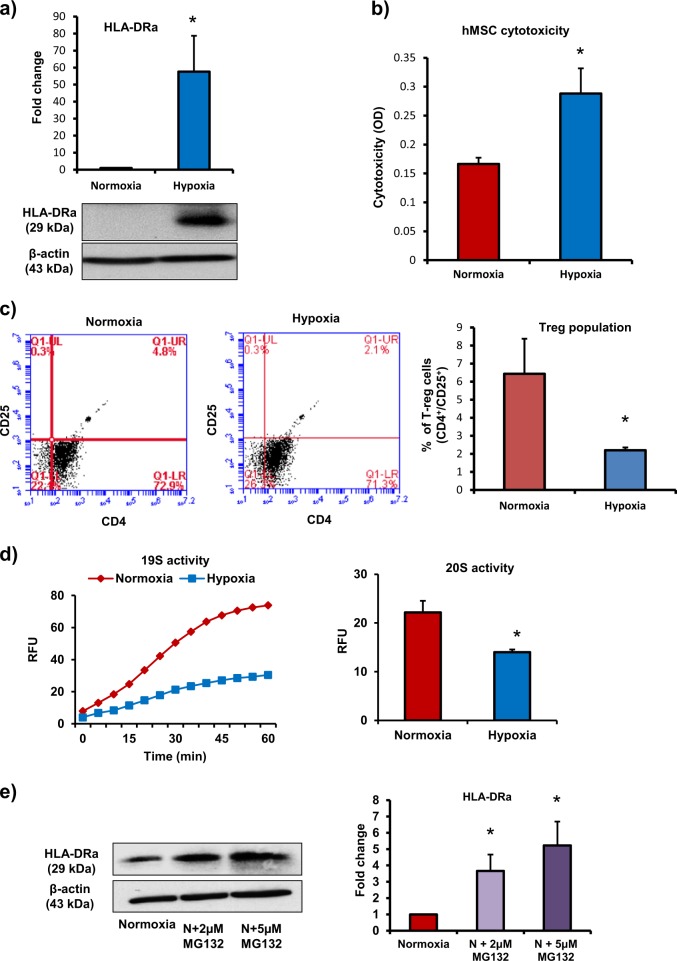
Loss of immunoprivilege of human MSCs after exposure to hypoxia. **a** Human bone marrow-derived MSCs (hMSCs) were exposed to hypoxia for 24 h. HLA-DRα levels as measured by western blotting increased in hypoxic MSCs (*n* = 3). **b**, **c** To determine the immunogenicity of MSCs, normoxic and hypoxic hMSCs were co-cultured with allogeneic leukocytes at a ratio 1:10 for 72 h. **b** Leukocyte-mediated cytotoxicity (LDH release) increased significantly in hypoxic hMSCs vs. normoxic cells (*n* = 10). **c** The effect of hMSCs on Treg cell (CD4^+^CD25^+^) induction in a mixed leukocyte population was assessed by flow cytometry. The number of Treg cells decreased after co-culture with hypoxic hMSCs (*n* = 4). **d** 26S proteasome activity was measured by determining the activities of both 19S (deubiquitinating activity) and 20S (proteolysing activity). The exposure to hypoxia led to a significant decrease in 26S activity in hMSCs (*n* = 4). **e** hMSCs were treated with 26S proteasome inhibitor (MG132, 2 µM and 5 µM for 24 h); HLA-DRα levels determined by western blotting showed a dose-dependent increase (*n* = 3); *p < 0.05 compared with normoxia group. Each experiment was repeated four to six times

**Fig. 8:**
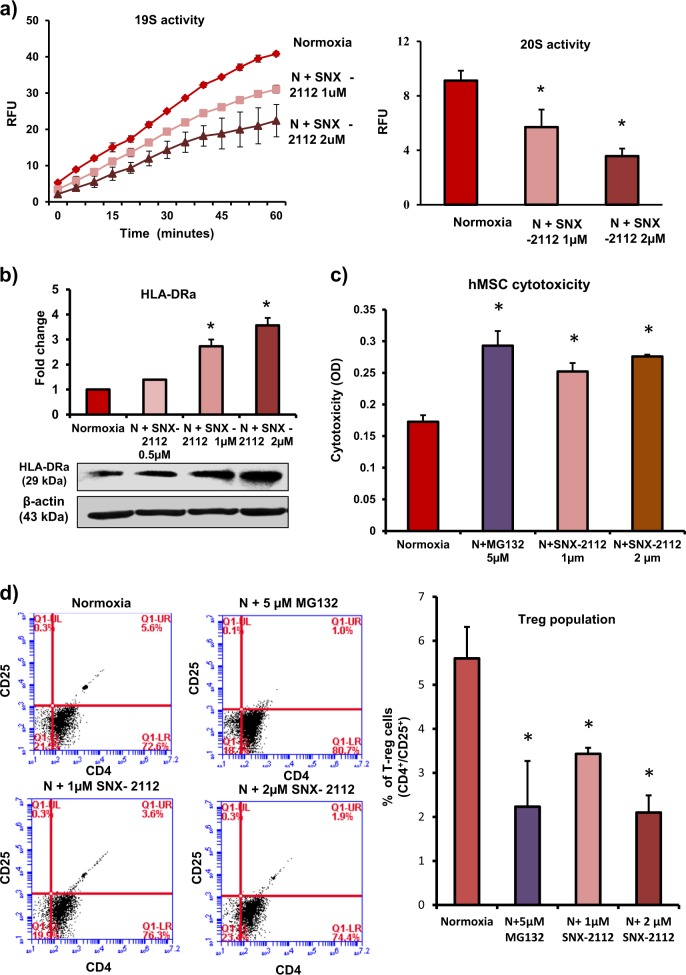
HSP90α maintains 26S activity and preserves immunoprivilege of hMSC. **a**, **b** hMSCs were treated with HSP90α inhibitor (SNX-2112, 0.5 µM, 1 µM, and 2 µM for 24 h), 26S activity (19S and 20S activities) by fluorescence assay, and HLA-DRα levels by western blotting were measured. **a** 26S activity decreased in HSP90α-inhibited MSCs (*n* = 3). **b** HLA-DRα expression increased in HSP90α-inhibited MSCs (*n* = 3). **c**, **d** To determine the immunogenicity of hMSCs after HSP90α inhibition, hMSCs were treated with SNX-2112 (0.5 µM, 1 µM, and 2 µM for 24 h) and then co-cultured with allogeneic leukocytes at a ratio 1:10 for 72 h. **c** Leukocyte-mediated cytotoxicity (LDH levels) in hMSCs increased significantly in the presence of HSP90α inhibitor (*n* = 10). **d** Treg (CD4^+^CD25^+^) cell number in the mixed leukocyte population decreased significantly after co-culture with HSP90α-inhibited hMSCs. *n* = 10. *p < 0.05 compared with normoxia group. Each experiment was repeated four to six times

To demonstrate translational potential of our studies, we also performed parallel experiments in human bone marrow-derived MSCs (hMSCs). In rodents, MHC-II, and in humans, the molecules of MHC-II complex, human leukocyte antigens-DR (HLA-DR), HLA-DP, and HLA-DQ present antigens to CD4^+^ T cells leading to activation and proliferation of T cells and allograft rejection^27,28^. Our data demonstrate that exposure to hypoxia led to loss of immunoprivilege in hMSCs. We found a significant increase in HLA-DR molecule in hypoxia-exposed hMSCs (Fig. 7a). In hMSCs and allogeneic leukocytes co-culture experiments, the level of cytotoxicity was significantly higher in hypoxia-exposed hMSCs compared with normoxic cells (Fig. 7b). In addition, there was a significant decrease in Treg cell number in the mixed leukocyte population after co-culture with hypoxic hMSCs compared with normoxic cells (Fig. 7c). Therefore, exposure to hypoxia was associated with loss of immunoprivilege in hMSCs. Furthermore, we found a significant decrease in 26S levels in hypoxia-exposed hMSCs vs. normoxic cells (Fig. 7d). The inhibition of 26S activity in normoxic hMSCs led to an increase in HLA-DR expression in a dose-dependent manner (Fig. 7e). Furthermore, the inhibition of HSP90α (using pharmacological inhibitor SNX-2112) in normoxic hMSCs resulted in a significant decrease in the 26S activity and an increase in HLA-DR protein levels (Fig. 8a, b). In the leukocytes and normoxic hMSCs co-culture experiments, the inhibition of 26S activity and HSP90α levels led to an increase in leukocyte-mediated cytotoxicity in hMSCs (Fig. 8c). The number of Treg cells in the mixed leukocyte population decreased after co-culture with 26S and HSP90α-inhibited normoxic hMSCs compared with control group (Fig. 8d). Therefore, in normoxic hMSCs, HSP90α maintains 26S proteasome function and immunoprivilege of cells. The exposure to hypoxia leads to inactivation of 26S proteasome, increase in HLA-DR, and loss of immunoprivilege.

## Discussion

The outcome of several allogeneic MSC-based preclinical studies and initial clinical trials suggested that bone marrow-derived MSCs have the potential to treat a number of degenerative diseases. However, the beneficial effects of transplanted allogeneic MSCs were short lived, which has caused decline in the overall enthusiasm about MSC therapy. In fact, recent analyses of allogeneic MSC-based studies demonstrated that cells after transplantation turned immunogenic and were subsequently rejected by host immune system^29–31^. A number of studies have reported the mechanisms of immunoprivilege of MSCs^10,12^. However, the mechanisms of immune switch in MSCs from immunoprivileged to immunogenic state have not yet been studied conclusively, understanding this would help in planning strategies to prevent rejection and preserve the benefits of allogenic MSC-based therapies. Here we demonstrate that immunoprivilege of MSCs is tightly mediated by the absence of MHC-II. We identified that MHC-II expression increased in both rat and human (HLA-DR) MSCs after exposure to hypoxia, which was associated with loss of immunoprivilege. We found that 26S proteasome-mediated degradation of ubiquitinated MHC-II protein in normoxic MSCs downregulated MHC-II expression and preserved immunoprivilege of MSCs. Our data demonstrate for the first time that hypoxic environment lead to inactivation of 26S proteasome and loss of immunoprivilege of MSCs. The 26S function in MSCs is maintained by a chaperon protein HSP90α; the levels of HSP90α decreased in hypoxic MSCs. Furthermore, maintaining HSP90α levels in MSCs prevented hypoxia-induced inactivation of 26S proteasome and preserved immunoprivlege of MSCs.

Hypoxia is the integral component of ischemic environment, which is associated with a majority of pathological conditions in the body. Several studies have investigated the effects of hypoxia on proliferation and differentiation potential of MSCs^13,32,33^, and reported that exposure to mild-to-moderate degree of hypoxia (3–21% of oxygen) increases proliferation and differentiation of MSCs^34^. However, exposure to severe hypoxic conditions (1% or < 1%) significantly decreases proliferation and differentiation of MSCs^35^. In the bone marrow, oxygen levels range between 4% and 7%; hence, bone marrow-derived MSCs are adapted to moderate hypoxic conditions^36^. However, in the ischemic tissues (where stem cell transplantation is required), the oxygen level drops below 1%, leading to severe hypoxia. The effects of such a low level of oxygen (severe hypoxia) on the immunoprivilege of MSCs are largely unknown and it requires thorough investigation to maximize the regenerative potential of MSCs. Our data demonstrate that exposure to severe hypoxic conditions lead to a transition from an immunoprivileged to immunogenic phenotype in MSCs.

Bone marrow-derived MSCs are considered to be immunoprivileged, mostly because they do not express MHC-II on the surface. MHC-II is expressed constitutively in antigen-presenting cells including dendritic cells and B cells^37,38^. However, this molecule can also be induced in other cell types by interferon (IFN)-γ stimulation^3,39,40^. In the current study, normoxic MSCs expressed negligible amount of MHC-II and the cells were originally immunoprivileged and induced negligible immune reaction. However, after exposure to hypoxia there was an increase in MHC-II levels and MSCs became immunogenic. Our results are in conjunction with other studies where MSCs, when exposed to any stress, e.g., treatment with IFN-γ and interleukin (IL)-1β, led to an upregulation of MHC-II, which was associated with the increase in immunogenicity of MSCs^39–41^.

The life cycle of MHC-II in the cells has been studied extensively—especially its synthesis, activation, transport, and storage^42^. However, the turnover of MHC-II protein itself remains largely unexplored. In this regard, 26S proteasome is reported to mediate degradation of unwanted or damaged proteins by proteolysis. Previously, the role of 26S proteasome in MHC-I antigen processing and presentation has been reported^43^. The inhibition of 26S proteasome can cause a decline in MHC-I antigen processing and presentation. Similarly, the antigen loading for MHC-II has been reported to be enhanced through poly-ubiquitination of MHC-II in the lyso-endosomal complexes^44^. However, to the best of our knowledge, the involvement of 26S proteasome in MHC-II molecule turnover has not been investigated yet. In the current study, inhibition of 26S proteasome function in normoxic MSCs led to an increase in MHC-II levels and immunogenicity of MSCs. The 26S proteasome assembly comprised two subunits: 20S core subunit and 19S regulatory subunit. These two subunits bind together to form an active proteasome complex and perform degradation of unwanted proteins. Our data demonstrate that exposure to hypoxia caused dissociation of 19S and 20S subunits, and downregulation of 26S proteasome activity in MSCs. Previously, it has been reported that intracellular oxidative stress leads to dissociation of 20S and 19S subunits of 26S proteasome^45^. The inactivation of 26S proteasome in the current study was associated with accumulation of ubiquitinated MHC-II and loss of immunoprivilege of MSCs. Furthermore, molecular chaperones are quintessential to the binding of subunits as well as complex formation of the 26S system. Thence, we investigated various chaperones having a direct role in the assembly of 26S system. HSP90α registered a marked decrease in MSCs under hypoxic conditions. HSP90α is among the most abundant proteins in the body^46^. The major role of HSP90α is to bind and fold other proteins into their functional three-dimensional structures. It is also reported to have a role in assembling of 26S proteasomal machinery^47,48^. However, the role of HSP90α in the immunoprivilege of MSCs has not been investigated yet. When we blocked HSP90α in normoxic MSCs, the cells became immunogenic. On the other hand, overexpression of HSP90α in hypoxic MSCs maintained 26S activity and preserved immunoprivilege of MSCs. Therefore, HSP90α downregulation in hypoxic MSCs is associated with inactivation of 26S proteasome and loss of immunoprivilege of allogeneic MSCs. Interestingly, some studies have previously reported an increase in HSP90α levels under hypoxic conditions. Almgren and Olson^49^ found upregulation of HSP90 in vascular336 tissue exposed to hypoxic environment. In H9c2 cells, treatment with CoCl_2_ (a hypoxia mimetic agent) at 50–200 μM concentrations prevented serum and glucose deprivation-induced decrease in HSP90^50^. Therefore, hypoxia-induced alterations in HSP90α seem to be cell specific as well as dependent upon dose and duration of hypoxic conditions.

The present study suggests that 26S proteasome-mediated degradation of MHC-II maintains the absence of MHC-II on MSC’s surface that preserves immunoprivilege of MSCs (Supplementary Figure 6). The exposure to hypoxia led to inactivation of 26S proteasome assembly and loss of immunoprivilege of MSCs. These observations provide unique insights into the mechanisms responsible for hypoxia-induced loss of immunoprivilege of MSCs. Our data also suggest that maintaining optimal level of HSP90α preserves the immunoprivilege of MSCs under hypoxic conditions. More significantly, our studies reveal that hypoxia-induced loss of immunoprivilege is not only limited to rodent MSCs but hMSCs are also susceptible to hypoxia-induced immune switch from immunoprivileged to immunogenic state. Further, we have shown in a definitive manner that therapeutic interventions are possible through genetic modification (overexpression) of HSP90α, which can be targeted to preserve immunoprivilege of MSCs under hypoxic conditions. Therefore, our study may help in increasing the success rates of ongoing allogeneic MSC-based clinical trials and allowing a better planning for future trials.

## Material and methods

### Experimental animals

Unrelated Sprague–Dawley rats were used for the isolation of MSCs from the bone marrow and for the isolation of splenic leukocytes. The study protocols were approved by the Animal Care Committee of the University of Manitoba and conformed to the “Guide for the Care and Use of Laboratory Animals” published by the US National Institutes of Health (NIH Publication No. 85-23, revised 1985).

### Rat MSCs isolation and characterization

Rat MSCs were isolated from the femurs and tibias as previously described^4,12^. After connective tissue around the bones was removed and both ends snipped, the bone marrow plugs were flushed with Dulbecco’s modified Eagle’s medium supplemented with 15% fetal bovine serum, 100 units/ml penicillin G, and 0.1 mg/ml streptomycin. Cells were plated and cultured in the same medium. Next day, the medium was changed and non-adherent cells were discarded. The medium was replaced every 3 days and the cells were sub-cultured when confluency exceeded 90%. To characterize the cells, flow cytometry was performed—the cells which were CD44^+^ and CD29^+^ (Santa Cruz), and negative for hematopoietic progenitors markers CD45^−^ and CD34^−^ (Santa Cruz) were used for further experiments^4,12^.

### Human mesenchymal stem cells

Bone marrow-derived hMSCs were purchased from Lonza (PT 2501 CA10064-080). All the human MSCs related in vitro studies were approved by the University of Manitoba’s Research Ethics Board.

### Experimental treatments

Hypoxia treatment was employed for 24 h and the culture plates were placed in hypoxia chamber (oxygen level regulated at 0.0–0.1%) in the incubator (Biospherix hypoxia chamber). To block 26S proteasome, MSCs were treated with its specific inhibitor MG132 (2 µM and 5 µM) for 24 h. To inhibit HSP90α activity in normoxic MSCs, the cells were treated with SNX-2112 (0.5 µM, 1 µM, and 2 µM) for 24 h.

### Western blotting

The protein levels for MHC-II, NOB1, BLM10, HSP90α, HSP90β, and HLA-DRα were measured by western blotting using species-specific antibodies. Briefly, total protein levels were measured by Bradford method and 40 μg of protein was loaded onto SDS-PAGE. Following electrophoresis, proteins were transferred to PVDF membrane and incubated with appropriate primary and secondary antibodies. The membranes were developed using X-ray film and bands were quantified using Quantity One software for densitometry.

### Immunoprecipitation

The IP procedures were carried out according to the manufacturer’s guidelines (Santa Cruz Biotechnology). Briefly, total cell lysates were prepared from the cells in different groups. The lysates were then precleared using appropriate preclearing matrix. To form IP antibody–IP matrix complex, 40–50 µl of suspended (25% v/v) IP matrix and 1–5 µg of IP antibody in 500 µl of phosphate-buffered saline (PBS) were incubated overnight at 4 °C. Three hundred micrograms of total cellular protein was transferred to the pelleted matrix and incubated overnight at 4 °C. The samples were then analyzed using electrophoresis as described for the western blotting procedure and probed with primary antibodies and secondary antibodies. The membranes were developed using X-ray film and bands were quantified using Quantity One software for densitometry.

### Two-dimensional blue-native PAGE assay

The 2DBN-PAGE/SDS-PAGE assay was performed to study the association of proteasome subunits 19S and 20S. The first dimension BN-PAGE and second dimension SDS-PAGE were performed as described previously^21^. Briefly, the cell lysates were prepared by sonication in 20 mM Bis-tris, 500 mM ε-aminocaproic acid 20 mM NaCl, 2 mM EDTA (pH 8.0), and Glycerol 10% supplemented with 1 × Halt protease and phosphatase inhibitor cocktail (Thermo Scientific), and 1.5% *n*-Dodecyl β-d-maltoside (Sigma). The proteins were then separated in 4–15% gradient blue-native polyacrylamide gel. The gel strips (individual lanes) were carefully excised including the 3.2% stacking gel and immersed in the Laemmli sample buffer containing freshly prepared dithiothreitol (54 mg/ml). The gel slices were incubated in sample buffer for 30 min at room temperature (RT) and then the proteins in the gel slices were separated in second dimension SDS-PAGE and immunoblotted using specific antibodies for Sug1 (one of the constituents of 19S subunit) and α3 (one of the constituents of 20S subunit).

### Immunocytochemistry

MSCs were seeded onto sterile coverslips and allowed to grow till 60% confluency. The plated cells were fixed with 4% paraformaldehyde and permeabilized using 0.2% Triton X in PBS at RT. The cells were then stained with respective primary and secondary antibodies, and phalloidin (for F-actin, Invitrogen). Thereafter, the cells were counter stained with DAPI (4′,6-diamidino-2-phenylindole) for nuclei. The cells were imaged using Cytation 5 imaging system (BioTek Instruments).

### Reverse-transcription PCR

Total RNA was isolated using high pure RNA isolation kit (Roche) and transcribed to complementary DNA using cDNA kit (Thermo scientific) for reverse transcription-PCR. The following PCR primers were used: BLM10- forward primer: 5′-CGTGTGGATGGGAAGAAGTT-3′, reverse primer: 5′-CAGAAGGCGGCTTGTTAAAG-3′; HSP90α forward primer: 5′-CAACCAATGGAGGAAGAGGA-3′, reverse primer: 5′-AGCGTCTGAGGAGTTGGAAA-3′; NOB1- forward primer: 5′-GATGGGTCTGAGAACCTGGA-3′, reverse primer: 5′-CTCCTCCCTTCCATCAATCA-3′; and HSP90β1- forward primer: 5′-GTCGGGAAGCAACAGAGAAG-3′, reverse primer: 5′-CTGGTATGCTTGTGCCTTCA-3′. The PCR products were loaded onto 1% agarose gel after mixing the samples with 6 × DNA loading buffer. The gels were imaged using ChemiDoc system (BIO-RAD).

### MHC-II siRNA inhibition in MSCs

We employed siRNA to block MHC-II in rat MSCs; for that we used siGENOME Rat RT1-Bb (Catalog number M-102315-00-0005) and as a control siGENOME siRNA (Catalog number 1D-001206-13-050) from Dharmacon. We used FuGENE® HD Transfection Reagent from Promega. Briefly, 100 µM stock solution of siRNA was prepared. One million MSCs were seeded per well and 80 pmol of both targeting and non-targeting siRNA were added after incubating the siRNA with Fugene HD for 10 min. This was followed by addition of siRNA-Fugene complex to each well and incubation in the CO_2_ incubator for 18 h. Next day, the cells were used to perform further experiments.

### 26S proteasome activity assay

To determine 26S proteasome activity, we measured deubiquitinating activity of 19S and proteolysing activity of 20S by fluorescent substrates. The deubiquitinating activity of 19S was measured by using Ubiquitin-Rhodamine 110 (Boston Biochem) at a concentration of 1 μM. The fluorescent intensity of each well was read at 485 nm (excitation) and 535 nm (emission) for 1 h with reading interval of 5 min. The 20S subunit activity was determined by a kit purchased from Cayman Chemicals (10008041).

### Mixed leukocyte-mediated cytotoxicity

To measure leukocyte-mediated cytotoxicity in rat MSCs, the leukocytes were isolated from spleen (SD rat) using HISTOPAQUE 1083 (Sigma-Aldrich) and co-cultured with allogeneic MSCs in the ratio of 10:1 as described in our previous studies^12,51^. After 72 h of co- culture, leukocyte-mediated cytotoxicity in the MSCs was assessed by measuring the LDH released from the damaged MSCs (LDH Cytotoxicity Detection Kit; Clontech).

To measure leukocyte-mediated cytotoxicity in human MSCs, hMSCs were co-cultured with leukocytes isolated from peripheral blood derived from healthy individuals at a ratio 1:10 for 72 h.

### Leukocyte proliferation

Leukocytes were co-cultured with allogeneic normoxic and hypoxic MSCs (10:1). The leukocyte proliferation was assessed after co-culture with MSCs by flow cytometric analysis (BD Accuri). Briefly, after 72 h of co-culture, the leukocytes in the supernatant were collected and centrifuged at 1000 r.p.m. for 5 min. The pellet was washed three times using PBS and suspended in 100 μl of cold PBS. After fixing with 5 ml of 70% ice-cold ethanol, the cells were treated with RNase (20 µg/ml) for 30 min. The leukocytes were then stained with propidium iodide (5 μg/ml) for 5 min at RT and analyzed using flow cytometry. To measure leukocyte proliferation, cell cycle analysis was done by counting the number of cells entering S-phase (proliferating phase) and G2/M phase from G0/G1 phase (resting cells) of the cell cycle. The leukocyte proliferation was also measured by a Cell Proliferation Assay Kit (Biovision Inc. Cat # K301).

### Treg cell measurement

The number of CD4^+^CD25^+^ Treg cells were counted in total leukocyte population after 72 h of co-culture with allogeneic MSCs using BioRad Treg cell estimation kits for rat (Catalog number DC040) and human (Catalog number DC027) by flow cytometry.

### HSP90α overexpression

Rat MSCs were transduced with lentiviral vector encoding the genes for Hsp90α and green fluorescent protein (GFP) (Vector builder, LVS-VB160907-1147sms) at 25 multiplicity of infection (MOI) for 24 h followed by second induction dose of 25 MOI the next day. The lentiviral vector encoding only GFP (empty vector: Vector Builder, LVL-VB160109-10005) was used as control. Transduction efficiency was assessed by fluorescence microscopy. To generate stable HSP90α-overexpressing MSCs, cells were grown under selection media containing Puromycin at a dose of 2.5 µg/ml. The HSP90α levels were assessed using western blot analysis.

### Statistical analysis

Experimental values are expressed as mean ± SD. Comparison of mean values between various groups was performed by one-way-analysis of variance followed by multiple comparisons by Tukey’s test. *P*-value < 0.05 was considered to be significant.

## Supplementary information

Supplementary Data
