# Supplementary material for: Hypoxia-induced 26S proteasome dysfunction increases immunogenicity of mesenchymal stem cells
Source: Cell Death Dis. 2019 Jan 28;10(2):90. doi: 10.1038/s41419-019-1359-x (PMC6349874; doi:10.1038/s41419-019-1359-x)

Supplementary figure 1: Blocking MHC-II (with or without 26S inhibition) in normoxic MSCs does not affect immunoprivilege. Rat bone marrow derived normoxic MSCs (with or without siRNA against MHC-II; and MG132 at 5 μM) were co-cultured with allogeneic leukocytes at a ratio 1:10 for 72 h. Leukocyte mediated cytotoxicity (LDH release) did not show any significant change in normoxic MSCs vs. MHC-II inhibited normoxic MSCs vs. MHC-II inhibited MG132 treated normoxic MSCs. (n=8).

Supplementary figure 2: Blocking MHC-II in hypoxic MSCs downregulated allogeneic leukocyte proliferation. Rat bone marrow derived normoxic MSCs and hypoxic MSCs (with or without siRNA against MHC-II) were co-cultured with allogeneic leukocytes at a ratio 1:10 for 72 h. The effect of MSCs on leukocyte proliferation was measured by proliferation assay kit (Biovision Inc.). The rate of leukocyte proliferation increased after co-culture with hypoxic MSCs compared to normoxic cells, siRNA mediated inhibition of MHC-II in hypoxic MSCs decreased leukocyte proliferation after the co-culture. (n=10). *$p<0.05$ compared to leukocytes co-cultured with normoxic MSC; #$p<0.05$ compared to leukocytes co-cultured with hypoxic MSCs.

Supplementary figure 3: MG132 dose response in MSCs. MSCs were treated with MG132 (26S proteasome inhibitor) at different concentrations 2μM, 5μM, 6μM, 8μM and 10μM for 24h. LDH release was measured to assess cytotoxicity caused by MG132 in MSCs. MG132 at a dose of 2μM and 5μM was found to be safe and effective. These concentrations were used for further experiments (n=8). *$p<0.05$ compared to normoxic MSC; #$p<0.05$ compared to normoxia +5μM of MG132 group.

Supplementary figure 4: 26S proteasome regulates MHC-II levels and preserves immunoprivilege of MSCs. Immunoprecipitation (IP) analysis was performed in rat normoxic

and hypoxic MSCs to determine the involvement of 26S proteasome in the degradation of MHC-II. IP data revealed a significant accumulation of ubiquitinated MHC-II protein in hypoxic MSCs. IP was performed with MHC-II antibody, and blotting was performed with polyubiquitin antibody. Left panel: IP; Right panel: lysate. (n=3).

Supplementary figure 5: Blocking 26S proteasome in normoxic MSCs increased leukocyte proliferation. Rat bone marrow derived normoxic MSCs (with or without MG132) were co-cultured with allogeneic leukocytes at a ratio 1:10 for 72 h. The effect of MSCs on leukocyte proliferation was measured by proliferation assay kit (Biovision Inc.). The rate of leukocyte proliferation did not change after co-culture with MHC-II inhibited MG132 (5µM for 24 h) treated normoxic MSCs compared to normoxic cells. However, presence of only MG132 (2 µM and 5µM for 24 h) increased leukocyte proliferation after the co-culture. (n=10). *$p<0.05$ compared to leukocytes co-cultured with normoxic MSCs.

Supplementary figure 6: Schematic diagram depicts MHC-II regulation and immunoprivilege of MSCs. In normoxic MSCs MHC-II degradation by 26S preserves immunoprivilege of cells. Exposure to hypoxic environment leads to inactivation of 26S proteasome, and accumulation of MHC-II and loss of immunoprivilege of allogeneic MSCs.

Supplementary Figure 1

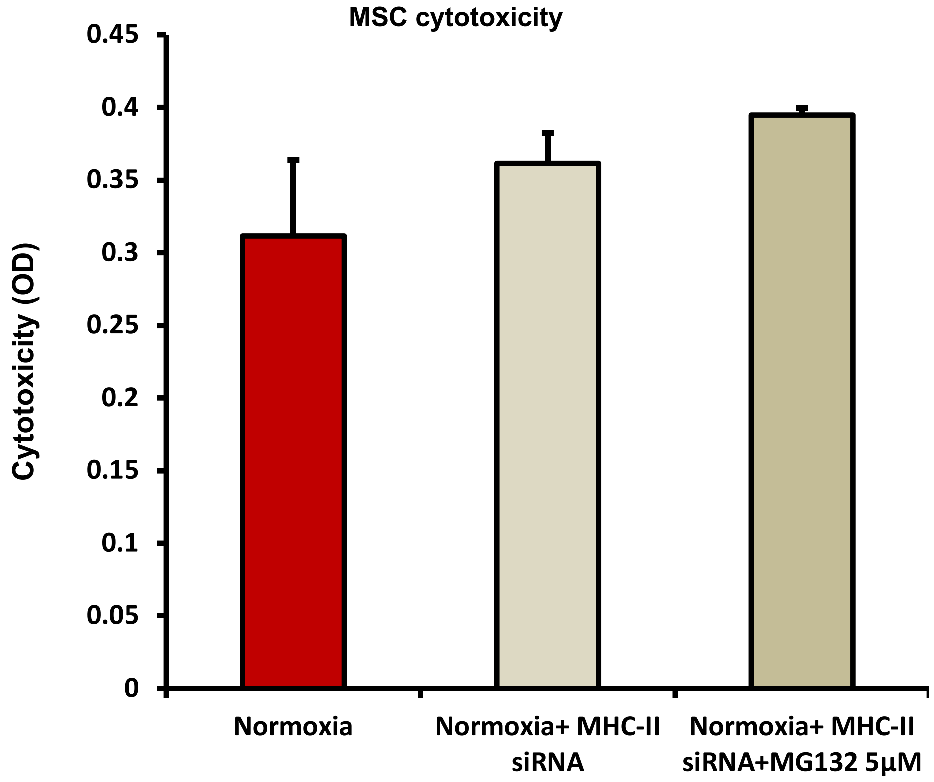

Supplementary Figure 2

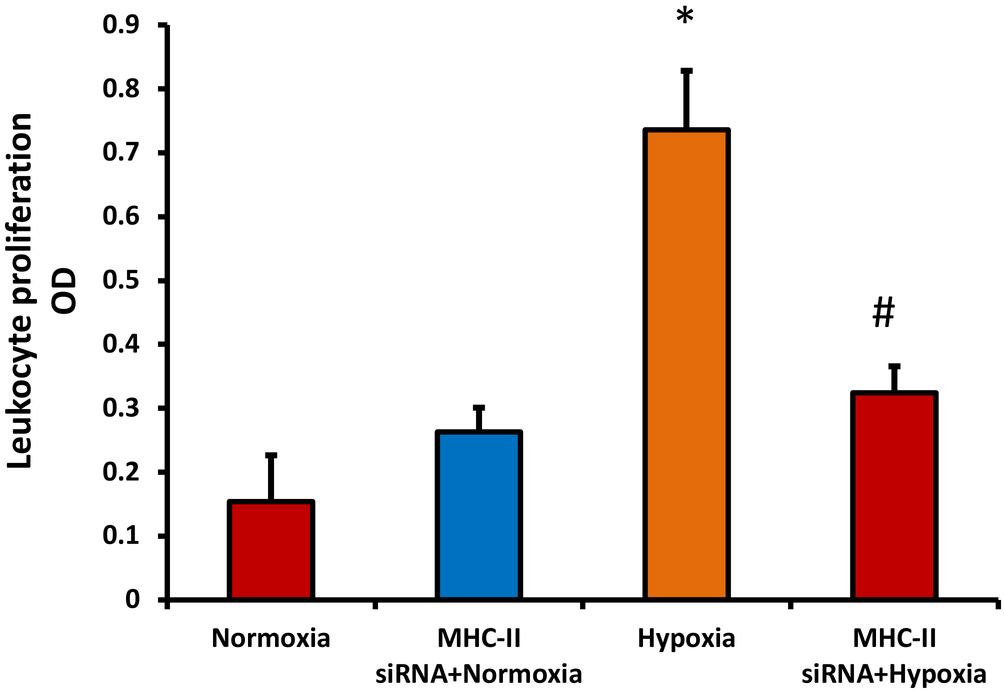

Supplementary Figure 3

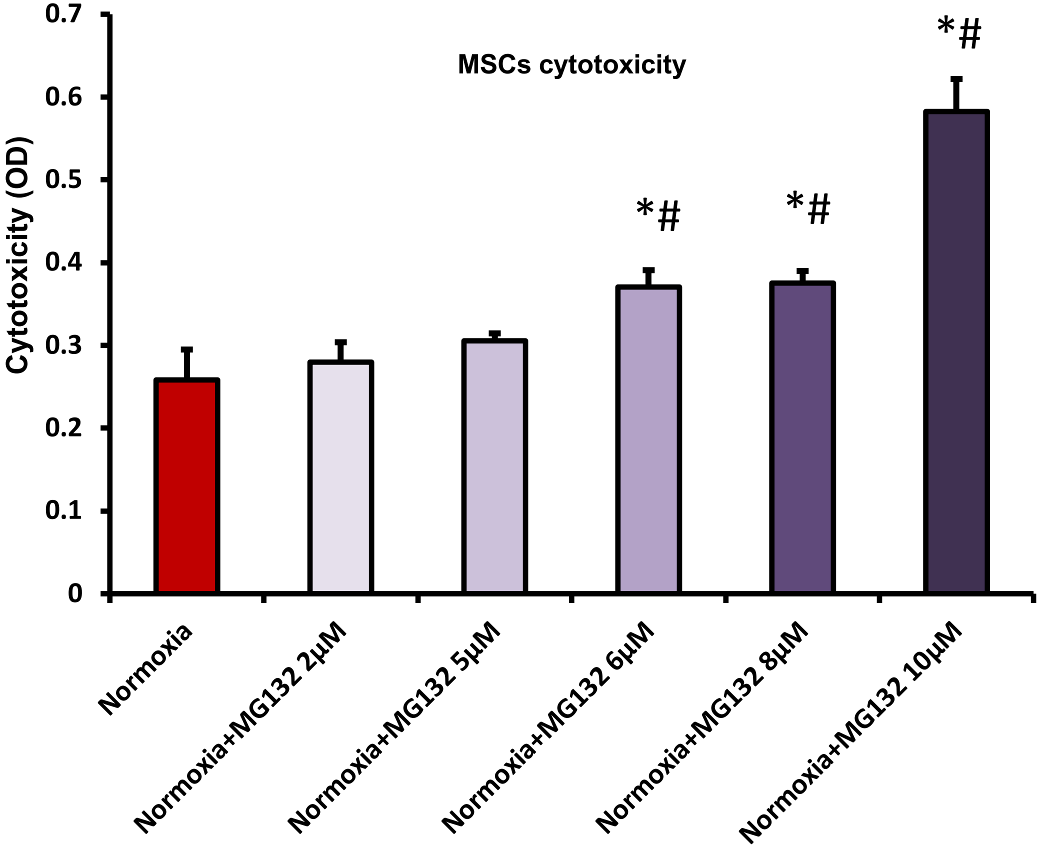

Supplementary Figure 4

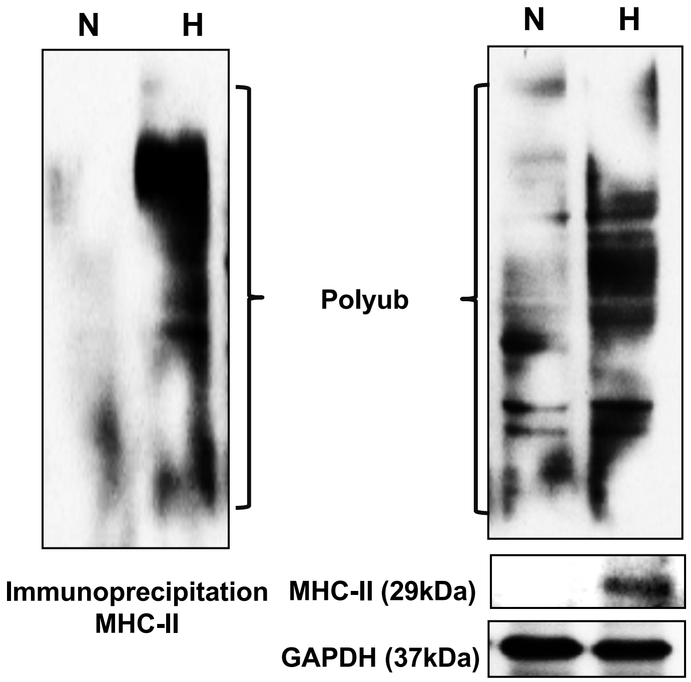

Supplementary Figure 5

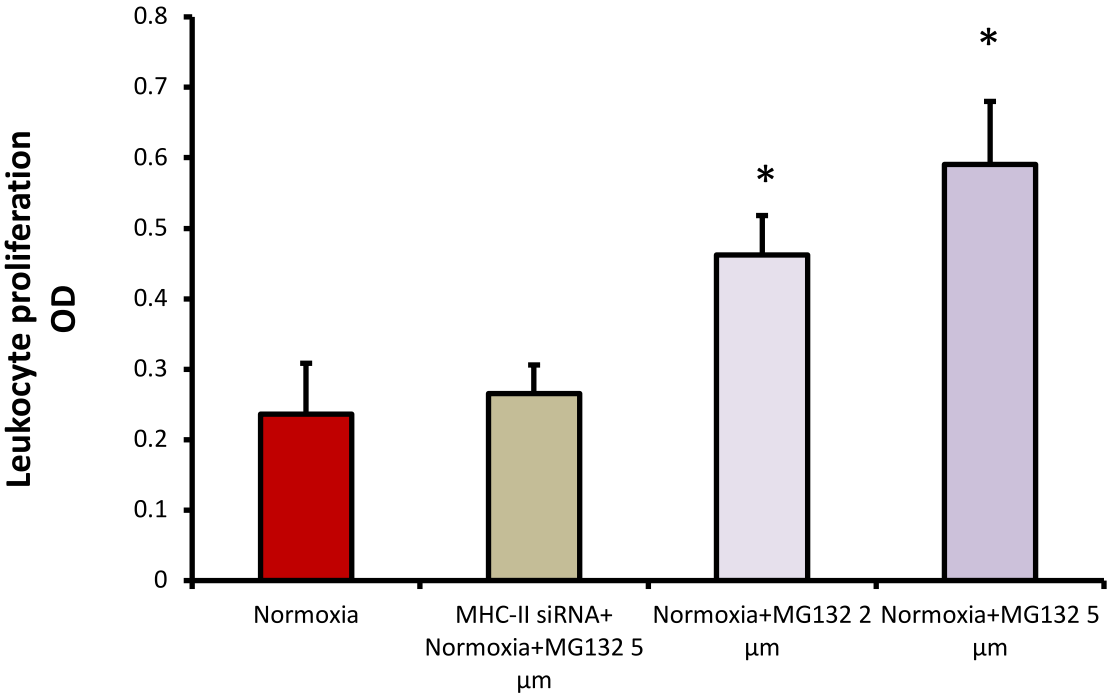

Supplementary Figure 6

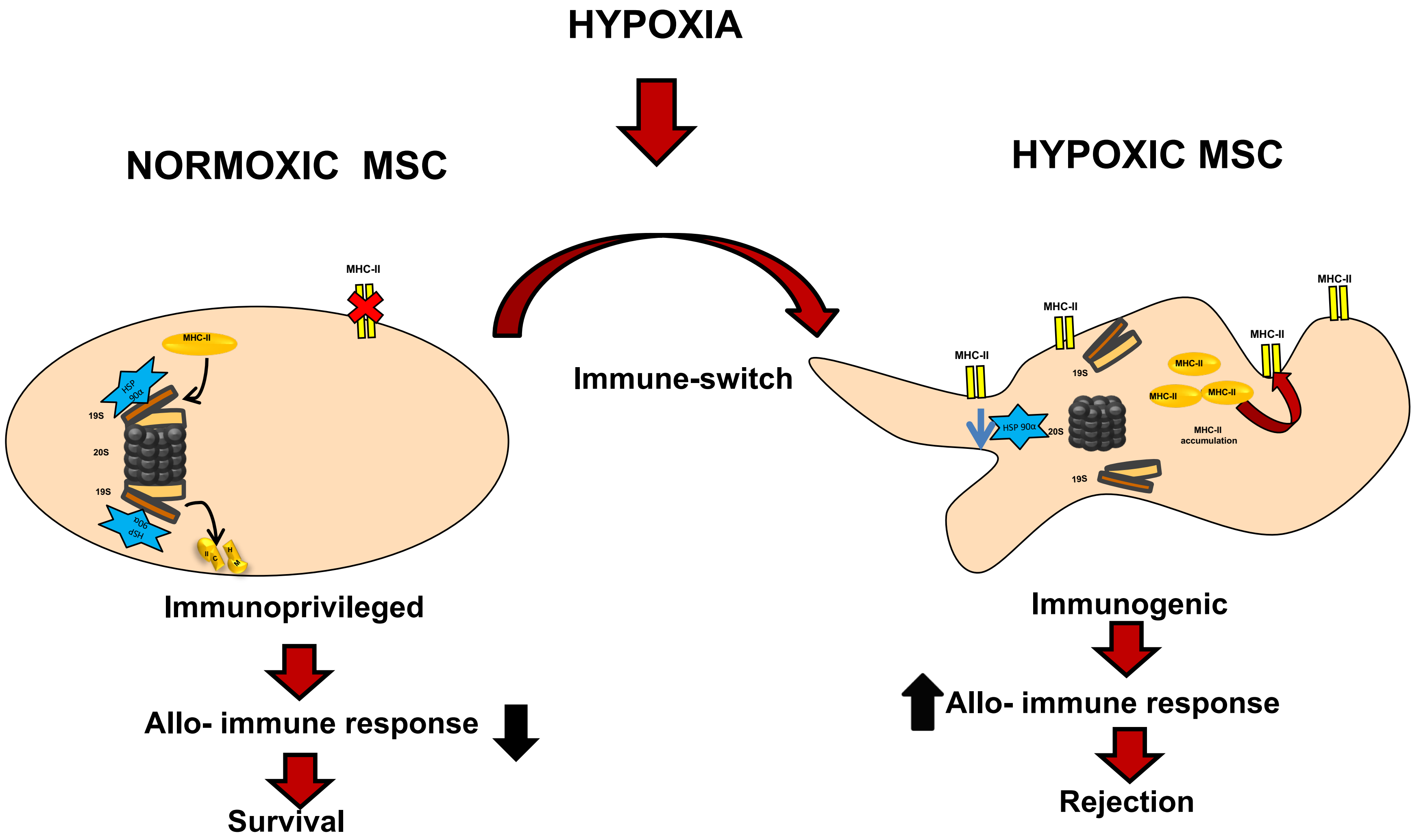

Supplement: Supplementary file 1 — Supplementary Data [file 41419_2019_1359_MOESM1_ESM.pdf]
